# Clinical Use and Adverse Drug Reactions of Linezolid: A Retrospective Study in Four Belgian Hospital Centers

**DOI:** 10.3390/antibiotics10050530

**Published:** 2021-05-04

**Authors:** Hélène Thirot, Caroline Briquet, Frédéric Frippiat, Frédérique Jacobs, Xavier Holemans, Séverine Henrard, Paul M. Tulkens, Anne Spinewine, Françoise Van Bambeke

**Affiliations:** 1 Pharmacologie Cellulaire et Moléculaire, Louvain Drug Research Institute, Université Catholique de Louvain, B1200 Brussels, Belgium; helene.thirot@uclouvain.be (H.T.); paul.tulkens@uclouvain.be (P.M.T.); 2 Clinical Pharmacy, Louvain Drug Research Institute, Université Catholique de Louvain, B1200 Brussels, Belgium; severine.henrard@uclouvain.be (S.H.); anne.spinewine@uclouvain.be (A.S.); 3 Groupe de Gestion de L’antibiothérapie, Cliniques Universitaires Saint-Luc, Université Catholique de Louvain, B1200 Brussels, Belgium; caroline.briquet@uclouvain.be; 4 Infectiologie, Centre Hospitalier Universitaire de Liège, Université de Liège, B4000 Liège, Belgium; f.frippiat@chuliege.be; 5 Infectious Diseases Department, Cliniques Universitaires Erasme, Université libre de Bruxelles, B1070 Brussels, Belgium; Frederique.Jacobs@erasme.ulb.ac.be; 6 Grand Hôpital de Charleroi, Infectiologie, B6000 Charleroi, Belgium; Xavier.HOLEMANS@ghdc.be; 7 Institute of Health and Society (IRSS), Université Catholique de Louvain, B1200 Brussels, Belgium; 8 CHU UCL Namur, Pharmacy Department, Université Catholique de Louvain, B5530 Yvoir, Belgium

**Keywords:** linezolid, adverse drug reaction, thrombocytopenia, anemia, serotonin syndrome, neuropathy, lactic acidosis, off label use

## Abstract

In Belgium, linezolid is indicated for pneumonia and skin and soft tissue infections, but is more broadly used, due to its oral bioavailability and activity against multiresistant organisms. This could increase the risk of adverse drug reactions (ADR), notably hematological disorders (anemia, thrombocytopenia), neuropathy, or lactic acidosis. We analyzed linezolid clinical use in relationship with occurrence of ADR in Belgian hospitals and highlighted risk factors associated with the development of thrombocytopenia. A retrospective analysis of electronic medical records and laboratory tests of adult patients treated with linezolid in four Belgian hospitals in 2016 allowed the collection of ADR for 248 linezolid treatments. Only 19.7% of indications were in-label. ADR included 43 thrombocytopenia, 17 anemia, 4 neuropathies, and 4 increases in lactatemia. In a multi-variate analysis, risk factors of thrombocytopenia were a treatment duration > 10 days, a glomerular filtration rate < 60 mL/min, and a Charlson index ≥ 4. Off-label use of linezolid is frequent in Belgium, and ADR more frequent than reported in the summary of product characteristics, but not statistically associated with any indication. This high prevalence of ADR could be related to a high proportion of patients presenting risk factors in our population, highlighting the importance of detecting them prospectively.

## 1. Introduction

The antibiotic linezolid was approved in the beginning of the year 2000. As first marketed representative of the oxazolidinone class, it opened new opportunities for the treatment of infections by Gram-positive bacteria, its mechanism of action being different from that of the other antibiotics acting on bacterial protein synthesis. Indeed, linezolid targets the peptidyl transferase center of the bacterial ribosome, causing preferential arrest of translation at specific sites and redistribution of the ribosomes on mRNA [1]. The indications reported in the Summary of Product Characteristics (SmPC) in European countries include exclusively community-acquired and nosocomial pneumonia as well as complicated and uncomplicated skin and skin structure infections [2]. The US label [3] adds to these indications the infections attributable to vancomycin-resistant *Enterococcus faecium* (VRE). However, linezolid presents a series of advantages that could incite the clinicians to use it in a larger number of circumstances. Indeed, linezolid is active against multiresistant Gram-positive organisms like methicillin-resistant *Staphylococcus epidermidis* or *S. aureus* (MRSE, MRSA) and VRE. Moreover, it shows a maximal oral bioavailability and large tissue distribution [4]. It is therefore a useful option when a switch from intravenous therapy (with linezolid or another drug like vancomycin) to oral treatment is possible to reduce the duration and cost of the hospitalization or to avoid renal toxicity related to vancomycin. It has also shown to be of interest in the treatment of difficult-to-treat infections by multiresistant pathogens, like endocarditis [5] or osteomyelitis [6]. Despite these advantages, linezolid usage is limited by rare but severe adverse drug reactions (ADR), namely hematological toxicity (thrombocytopenia, anemia), risk of developing peripheral and optic neuropathy [7,8] or, rarely, lactic acidosis [9,10]. Linezolid is also an inhibitor of monoamine oxidases, putting the patients at risk of developing a serotonin syndrome if it is associated with other drugs presenting the same risk [11]. In the registration clinical trials referred to in the SmPC or in the US label, the frequency of these ADR is either low or not well described [12,13]. ADR are rare but the presence of risk factors seems to increase the risk of developing them. The most frequently reported risk factors, especially for thrombocytopenia, are a long treatment duration [14], a bad renal function [15,16], and a low body weight [17,18]. In spite of these drawbacks, off-label use of linezolid is quite common [19,20,21].

Yet, it has been suggested that the off-label use of drugs could be associated with a higher risk of ADR [22]. Off-label use consists in using a drug in conditions that are not described in the authorized product information in terms of dose, route of administration, treatment duration, indication, or target patients’ population [23]. Pharmacovigilance good practices recommend to collect and report data about possible ADR related to off-label use [24], because these conditions of usage are new and may result in toxicity issues that were not highlighted during the registration clinical trials. Current reports that examined linezolid safety in relation with its local conditions of use (including off-label indications) are scarce and concern Japanese patients [14,15,16] or, in Europe, hematological toxicity in a single hospital in Spain (see for example [25,26]). A meta-analysis also compiled 12 studies from 11 countries and focused on safety of linezolid in patients with TB [27]. We are thus critically lacking of data considering more globally the safety profile of linezolid in its actual conditions of use in a European country.

The primary objective of this study was to assess the clinical use of linezolid in relationship with its toxicity in the Belgian hospital daily clinical practice. To reach this goal, we characterized in-label vs. off-label indications and the occurrence of ADR compared to safety reported in SmPC/US label. A secondary objective was to identify risk factors associated with the development of thrombocytopenia, the most frequently reported ADR in our study population.

## 2. Results

### 2.1. Study Subjects

In total, 230 patients met the inclusion criteria over the study period, representing 248 treatments (16 patients received two or three courses of linezolid therapy in the same year, usually for prosthetic joint infections). The general characteristics of the whole patient population are summarized in Table 1. Among the population, 37.1% were male. The median age is 65.

Inpatients showed a significantly lower renal function, shorter treatment duration, and lower basal platelet counts, but no difference in their Charlson index [28] as compared to outpatients (Table S1 (Supplementary Materials)).

### 2.2. Linezolid Treatments: Characteristics

The indications of linezolid and their relative proportions are described in Table 2.

Linezolid was used in-label according to the indications of the SmPC in European countries [2] (SSTI and pneumonia) in 19.7% of the cases only. A non-negligible proportion of the treatments exceeded 28 days (N = 35, 14.1%), including for some in-label SSTI (N = 4), and more frequently for bone and joint infections (N = 20) and endocarditis (N = 3). 

Indications were also variable among the participating hospitals (Figure S1 (Supplementary Materials)). Posology was in all cases 600 mg twice daily. Linezolid was administered intravenously in 101 patients and orally in 155 patients (with an IV to oral switch in 8 patients).

The most frequently isolated pathogens were *Enterococcus faecium* (25.7%), methicillin-resistant *Staphylococcus epidermidis* (23.7%), methicillin-resistant *Staphylococcus aureus* (17.5%), and vancomycin-resistant *Enterococcus* principally *faecium* (9.3%). Of the various cases, 67% of pneumonia cases were caused by methicillin-resistant *S. aureus*, 52% of bone and joint infections by methicillin-resistant *S. epidermidis*, 67% of gastrointestinal infections by *E. faecium* (out of which 5 are vancomycin-resistant *Enterococcus*), and 62% of urinary tract infections by *E. faecium* (Figure S2 (Supplementary Materials)).

**Figure 1:**
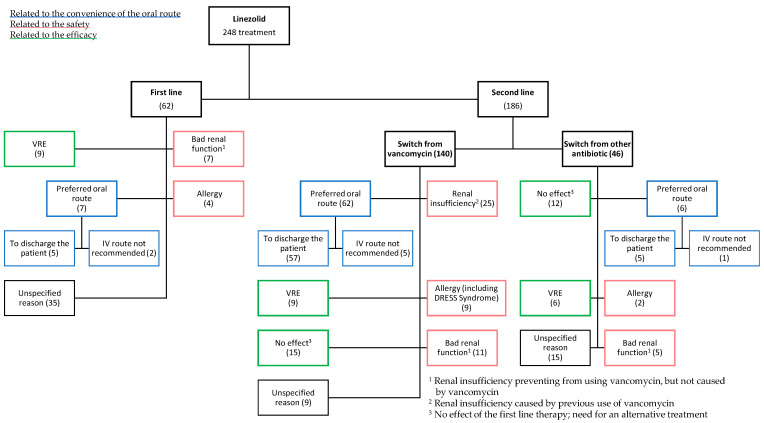
Flowchart: Reasons for prescribing linezolid. The color of the box defines the type of reason: convenience of oral route (blue), safety (red); efficacy (green).

Linezolid was prescribed as first and second line anti-infective therapy in 25% and 75% of cases, respectively (Figure 1). The main reasons for choosing linezolid (whether as first or second line) can be classified in three categories: oral route convenience, safety issues with other drugs, or efficacy. The oral route was chosen to discharge patients and shorten the duration of the hospitalization (27%) or because the intravenous line had to be removed (3.2%).

Safety reasons included: (1) renal insufficiency caused by vancomycin (10.1%), (2) renal insufficiency unrelated to vancomycin or avoiding the use of vancomycin by fear of toxicity (9.2%) and (3) allergy principally including DRESS syndrome (6%). Lastly, linezolid was selected in case of inefficacy of the first-choice drug, based on microbiological criteria (isolation of a vancomycin-resistant microorganism (9.7%)) or clinical criteria (absence of improvement despite antibiotic treatment (10.8%)). When prescribed as first line, reasons were principally the safety and the effectiveness of the treatment. When prescribed as second line, it was after vancomycin in most of the cases (139 patients; 75% of the second line), to allow switch to oral route or because of safety issues with vancomycin (renal insufficiency, or allergy). Among the other 25% of second line prescriptions, the first line drug was a β-lactam (18.5%; broad spectrum in most of the cases) or an antibiotic with a broader spectrum than linezolid (6.5%; tigecycline, clindamycin, moxifloxacin, trimethoprim/sulfamethoxazole). A complete analysis of the reasons for prescribing linezolid as first line and second line is illustrated in Figure 1.

### 2.3. Adverse Drug Reactions

At least one ADR was reported in the medical file of 39 (15.7%) patients (without considering ADR detected based on laboratory tests as anemia and thrombocytopenia), with a number of ADR per patient of 1 in 21 patients, 2 in 10 patients, 3 in 5 patients, and 4 in 3 patients, respectively. The treatment was discontinued in 16 patients (6.45%) because of toxicity (7 for thrombocytopenia, 4 for hypersensitivity, 2 for serious intestinal disorders, 1 for a suspicion of serotonin syndrome, 1 for increase in plasma lactate level, 1 for acute renal failure). The calculated Naranjo score for each of these ADR (see Methods; Ref. [29]) shows that they were possibly (i.e., score of 1–4) to probably (i.e., score of 5–8) associated with linezolid (Table 3).

The proportion of ADR was not statistically different between patients treated for in- or off-label indications, with 8 patients having developed an ADR (16%) among those treated for in-label infections (49 patients), vs. 31 patients (15.6%) among those treated for off-label indications (199 patients). 

Thrombocytopenia was the most frequent ADR, with 20 cases notified in the medical records but a total of 43 cases detected based on values from blood tests. Thrombocytopenia appeared after a median of 10 days. It was as frequently observed in inpatients and outpatients (Table S1 (Supplementary Materials)), but the time of onset was shorter in inpatients (10 days) compared to outpatients (15 days) [*p*-value = 0.009]. Charlson comorbidity index was associated to an earlier development of thrombocytopenia if ≥5, with patients with a value ≥ 5 developing thrombocytopenia earlier (10 days) than those with a value ≤ 5 (15 days) [*p*-value = 0.032]. The severity of the thrombocytopenia was variable among patients, with 2 patients in grade 4 (<25,000 platelets/mm^3^), 10 patients in grade 3 (25,000–50,000 platelets/mm^3^) and 8 patients in grade 2 (50,000–75,000 platelets/mm^3^). The remaining 23 patients with thrombocytopenia had levels comprised between 75,000 and 150,000 platelets/mm^3^.

Likewise, 9 cases of anemia were notified but 17 were identified from biological data, in similar proportions in inpatients and outpatients (Table S1 (Supplementary Materials)). Among these cases, 9 patients developed both thrombocytopenia and anemia. Anemia appeared after a median of 16 days of treatment with linezolid, with no statistical difference between inpatients and outpatients.

Among gastrointestinal disorders category, diarrhea (N = 2), nausea (N = 2), vomiting (N = 3), and loss of appetite (N = 6) were notified. 

Four patients developed peripheral neuropathy. Two of them received linezolid for a longer than recommended duration, i.e., 90 days and 84 days, respectively. The third patient received the drug during 22 days and the last one during 15 days but this patient was previously treated with linezolid during 52 days 2 months earlier. Two of these patients received an antiepileptic drug to relieve the peripheral neuropathy. 

Four patients developed an increase in lactatemia notified in their EMR (Reported values: 3.3, 4.1, 5.2, and 33.8 mmol/L, respectively [normal range: 0.5 and 2.2 mmol/L]). Among these patients, one took metformin at the same time. After the cessation of the treatment, values returned to normality.

Among concomitant treatments, at least one drug increasing the risk of developing a serotonin syndrome was noted in the medical files of 97 patients (39%), namely tramadol (53) or antidepressant drugs (Serotonin Selective Reuptake Inhibitors (SSRI) (23), trazodone (15), Serotonin-Norepinephrine reuptake inhibitors (11), mirtazapine (11), and tricyclic antidepressants (TCA) (5)). Based on the SmPC, linezolid is contraindicated in patients taking those kinds of drugs concomitantly, especially in the absence of close monitoring of serotonin syndrome signs. Despite the high level of comedication with molecules considered as contraindicated, only one clinical evidence of serotonin syndrome was declared in a patient taking trazodone and duloxetine at the same time as linezolid. Symptoms were delirium and agitation that disappeared a few days after interrupting linezolid treatment.

### 2.4. Factors Associated with Thrombocytopenia

Among the 248 treatments, 228 medical files contained complete data from blood samples. These 228 data sets were used for the analysis of parameters associated with the development of thrombocytopenia (Table S2 (Supplementary Materials)). Table 4 shows results from the univariate and the multivariate analysis. 

In the multivariate analysis, factors significantly associated with thrombocytopenia were: Charlson comorbidity index ≥ 4 (OR = 2.534 [1.128–5.694]), glomerular filtration rate < 60 mL/min (OR = 3.694 [1.649–8.275]), and treatment duration > 10 days (OR = 7.944 [3.197–19.738]). The comorbidities identified in patients with thrombocytopenia are detailed in Table S4 (Supplementary Materials).

## 3. Discussion

Our study shows that the off-label use of linezolid is frequent in Belgian clinical practice and that the prevalence of ADR is much higher than what is described in the SmPC in Europe or in the US label (Table S3 (Supplementary Materials)). Thrombocytopenia was the most frequent, with a prevalence of 18.9% (compared to ≥1/1000 to <1/100 in SmPC or 3% in the US label). Three factors were identified as being associated to thrombocytopenia, namely a high Charlson index, renal failure, and a long treatment duration.

Concerning first the infections treated, we found that linezolid use covers a broader panel of indications than those specified in the SmPC in Europe (off-label use: 80.3%). In our cohort, off-label use concerns principally the indications and the treatment duration. Interestingly, marked differences in these indications are observed among the participating hospitals, probably unveiling discrepancies in local recommendations. This situation is not unique to Belgium. As an example, a French study assessing the appropriateness of linezolid prescriptions in three hospitals found that, among 81 treatments, 65% were considered off-label regarding the indications [30]. A second study performed in a single French hospital recorded an off-label use of 45% between 2009 and 2013 [21]. A similar proportion (40%) was described in a monocentric Spanish study [31]. Linezolid is not the only antibiotic with reports of frequent off-label use. In a systematic review from 2012, the percentage of off-label use of antibiotics varied from 19 to 43% in adult critical-care patients [32]. In a prospective study, 31.2% of antibiotic prescriptions in a French tertiary hospital were off-label regarding the indications, which was attributed to a lack of update of market authorizations with new guidelines and a lack of investment of industries to extend their market authorizations for drugs that are often generic [33]. These arguments can also apply to linezolid.

Off-label does not necessarily mean that prescribing is inappropriate. First, the majority of bacteria isolated in our cohort of patients were susceptible to linezolid (except one *Enterococcus* that was discovered resistant to linezolid after three days of treatment and for which the therapy was switched to tedizolid). Second, some of the indications evidenced in our study are part of those accepted by the FDA (VRE infections) or recommended as second line therapy by the Infectious Disease Society of America (persistent bacteremia, central nervous system infections, and osteomyelitis caused by MRSA) [34]. Based on these American criteria, off-label use in our population would concern 60% of the prescriptions, a smaller but still important value. At the end, among off-label indications, 21.6% of the prescriptions may be considered as inappropriate as no clear reason for choosing linezolid (need for oral route, safety or efficacy reasons) was described or because of linezolid resistance (in a single isolate). For the rest (78.4%), acceptable reasons are specified, like oral administration and switch from IV route, activity against strains resistant to first-line drugs, or switch from vancomycin in case of renal insufficiency. 

A question arising related to this large off-label use is the potentially associated risk of developing ADR [22]. In a previous study, among 1488 adult inpatients who received a systemic antibiotic, 20% developed at least one ADR, among which 20% were associated to an antibiotic used off-label [35]. In our analysis, the prevalence of ADR was not statistically influenced by the indication, nor by the fact that the indication was in- or off-label. Although the number of prescriptions we analyzed is limited, our conclusion remains coherent with that of a study considering a large cohort of more than 46,000 patients receiving all types of drugs, for whom higher rates of ADR were observed for off-label prescriptions that specifically lacked strong scientific evidence [22]. This was not the case here for linezolid, since the majority of prescriptions remained appropriate.

The most striking difference in prevalence of ADR was noticed for thrombocytopenia, which concerned 18.9% in our population vs. 0.1 to 1% in the SmPC. Other studies also show high percentages of thrombocytopenia, with values ranging from 17 to 57% [14,15,16,17,18,25,36,37,38]. The way thrombocytopenia is defined may partly explain this disparity. We defined it as a reduction in platelet counts ≥ 30% from baseline and a value ≤ 150,000 cells/µL while other studies considered a decrease of 25% in platelet counts [25] or a decrease in platelet counts ≥ 50% from baseline [17]. The reasons why this prevalence is more than 20 times higher than that reported in the SmPC are possibly related to the high proportion of patients presenting associated risk factors in our cohort. We identified a Charlson index ≥ 4, a glomerular filtration rate < 60 mL/min, and a treatment duration > 10 days as significantly increasing the risk of developing thrombocytopenia. We may have identified Charlson index here because of the large proportion of patients presenting comorbidities in our population. Charlson index has been cited as a risk factor in only one study, combined to patient age [36]. Renal failure has already been described as a risk factor in previous studies [14,15,16,17,37]. Our cut-off value for renal failure was 60 mL/min as in Hanai’s [15] and Nukui’s [16] studies, but others described lower values, between 30 and 50 mL/min [14,25,38]. The renal function is also included in the Charlson index, but based on serum creatinine levels, with a cut-off value set at 3 mg/dL. This may represent a higher degree of renal insufficiency than a glomerular filtration rate < 60 mL/min and therefore concern a lower proportion of patients. Lastly, a treatment duration > 14 days is a demonstrated risk factor [14]. A surprising observation in our study is that patients with the longest treatment (> 55 days) did not develop thrombocytopenia. A probable explanation is that all these patients had a glomerular filtration rate > 60 mL/min and a Charlson index < 4. This observation indicates that the risk becomes more important when all risk factors are present. However as previously shown, linezolid is often used as an alternative in patients who developed renal failure potentially caused by vancomycin. This demonstrates that the implication of renal function in the toxicity of linezolid is still poorly known.

Other risk factors have been described in the literature, which were not highlighted in our study, like a low body weight [17,18], a low basal platelet count [18,25], or an older age [17]. The discrepancy may reside in the differences in the selected population. First, we chose to include all treated patients while other studies exclude patients treated with anticancer drugs [18,39] or hospitalized in intensive care unit (ICU) [25] or treated for less than seven days [15]. Noteworthy in this respect, the proportion of patients developing thrombocytopenia or anemia in our cohort was not higher among hospitalized patients or those receiving anticancer chemotherapy than among others. Second, most of the studies looking at factors associated with the development of thrombocytopenia were performed in Asia, where the morphometric parameters of the patients are highly different from Europe. As an example, the percentage of patients with a BMI < 20 in our study (9.6%) is lower than in studies reporting low body weight as a risk factor (33%) [17]. 

Predictive scores specifically developed to assess linezolid toxicity have been proposed, which take into account some of these risk factors. The first one includes variables as a basal platelet count ≤ 90,000/mm^3^, renal failure (creatinine clearance below 50 mL/min), moderate or severe liver disease, and cerebrovascular disease as a way to predict thrombocytopenia risk [25]. The second one includes age-adjusted comorbidity index and the duration of therapy to assess the risk of developing ADR [36], which actually corresponds to the factors we identified here. Another type of described risk factor is related to the global exposure of the patients to the drug. Different studies conclude that keeping linezolid C_min_ between 2 and 7 mg/L is efficacious and safe [40,41]. As inter-individual variations in serum levels are frequent among patients, these data would plead for implementing therapeutic monitoring [42], which is not used in routine so far. 

Regarding the other ADR, anemia was as frequent (6.8%) as described in the SmPC or in the US label (1–10%). A similar prevalence of 10% was observed in a previous study [15], which identified as the only risk factor a long treatment duration. The frequency of peripheral neuropathy and lactic acidosis is indeterminate in the SmPC or in the US label. Our study found four patients with peripheral neuropathy and four patients with an increase in their lactic acid level, which is quite high for a limited patients’ population. This may be due to the presence of patients with multiple comorbidities and/or with profile differing from those included in registration studies in our population. Some ADR reported in the US label [3] (hypoglycemia, elevated blood pressure, seizures, or *Clostridium difficile* diarrhea) were not reported in the EMR of our patients. In this context, underreporting of ADR is problematic, as it may prevent updating drug labels. It is worth mentioning here that only 31 ADR potentially related to the use of linezolid have been reported in Belgium between 2002 and 2016 to the national pharmacovigilance center (Data comes from the VigiBase (WHO global database of individual case safety reports). This information comes from a variety of sources. The likelihood that the suspected adverse reaction is drug-related is not the same in all cases. The information does not represent the opinion of the World Health Organization). Possible reasons are the additional workload for the healthcare providers, but also the difficulty to attribute an ADR to a specific drug, especially in multimorbid or polymedicated patients. Probability scales do exist, like the Naranjo score we used here, but all criteria were not applicable because of missing data, so that we could not exceed a score > 7 defining a probable ADR. 

A total of 39% of the patients received at least one drug increasing the risk of developing a serotonin syndrome. However only one patient potentially presented this reaction. Publications concerning the serotonin syndrome and linezolid are generally limited to case-reports and the incidence remains unclear [11]. The major class concerned by this interaction is the class of antidepressants (as SSRI or TCA). These treatments are sometimes taken for a long time and cannot be stopped before the beginning of linezolid. As this syndrome generally develops in the hours after taking drugs that increase serotonin levels, a follow-up of the typical symptoms could be performed in patients at risk that begin linezolid.

Our study has several limitations. First, due the retrospective character of our analysis, we were facing missing information, like unreported ADR or lack of detailed information on patient’s characteristics (weight, medical history) or on the reported ADR (blood monitoring). Second, despite the multicentric design, we cannot guarantee that the data collected represent the real global use in Belgium as the four centers selected are all in the French-speaking part of the country. In addition, VRE infections are often epidemic and their number may thus be variable from one year to the other.

## 4. Conclusions

This work allowed us to describe for the first time linezolid use and toxicity in representative Belgian hospitals, which may fuel discussion within hospitals or among them in order to better define the conditions of use of this drug and setup initiatives to predict and avoid the risk of developing ADR, not only in Belgium, but also in other countries with similar usage. It shows that ADR are much more frequent than reported in the SmPC or US label, demonstrating the impact of daily life practice for the safety of drugs, which has been assessed only in well-defined populations and conditions of use in registration trials. The discrepancy is particularly high for thrombocytopenia, underlining the need for an increased vigilance in the follow-up of multimorbid patients, those with a moderate to severe renal failure, and those with a long treatment duration.

In a broader context, our study may also stimulate critical thinking on the way clinical trials for antibiotics are set up to obtain their registration vs. the need to which they should respond in the clinics in terms of indications. Additionally, it points to the role healthcare professionals may play in reporting adverse events in order to correctly assess the benefit-risk balance of drugs for individual patients, considering their specific risk factors.

## 5. Materials and Methods

This retrospective observational study was performed in 4 Belgian hospital centers practicing tertiary care: 3 university hospitals (respectively 945 beds, 858 beds, 925 beds) and 1 non-university hospital (1154 beds) selected as using linezolid in at least 40 patients/year. All adult (≥18 years) patients treated with linezolid for at least 1 day between January 2016 and December 2016 were identified through dispensing data from the hospital pharmacy. In Belgium, linezolid is always prescribed in the hospital setting and delivered by the hospital pharmacy. Some patients receive the entire treatment in the hospital, while others start their treatment at the hospital for a few days and then continue it at home. There were no exclusion criteria; all patients, whatever the treatment duration, were included for the data descriptive analysis. For the analysis of thrombocytopenia, only patients with platelets data were included.

The following data, available for all in- and outpatients, were extracted from the electronic medical record (EMR) by the main researcher (HT) (from the beginning to the end of the treatment): demographic data, comorbidities to calculate the Charlson comorbidity index [28] (calculated through the creatinine level of the first day of the treatment), body weight, renal function (creatinine level and glomerular filtration rate estimated with MDRD (Modification of Diet in Renal Disease)), hemogram (frequency of platelet counts determinations for inpatients: every 3–4 days approximately; for outpatients: every 7–10 days approximately), microbiological data, previous antibiotic treatment, type of infection and reason for linezolid prescription, dosage, treatment duration, route of administration, and hospitalization status of the patient (inpatients received the entire treatment in the hospital and outpatients were defined here as patients starting their treatment at the hospital for 2 or 3 days and continuing it at home). Comedications that could increase the risk of developing a serotonin syndrome [43] or thrombocytopenia [44] were also considered.

ADR related to linezolid and reported in the EMR were collected (description, time of onset, management, recovery) and the potential relationship between linezolid administration and each ADR was assessed using the Naranjo probability scale [29]. For inpatients, data were extracted from hospitalization reports and the daily visits reports depending on the hospitalization service. For outpatients, consultation reports and previous reports of the hospitalization were consulted.

Regarding hematological disorders, thrombocytopenia was defined as a reduction in platelet counts higher than 30% from baseline and a value less than 150,000 cells/µL, and anemia, as a higher than 30% decrease in hemoglobin level from baseline and a value lower than 12 g/dL. The time of onset of thrombocytopenia was defined as the day when the platelets count decreases higher than 30% from baseline and reaches a value less than 150,000 cells/µL. Hyperlactatemia was defined when values were higher than the normal range 0.5 and 2.2 mmol/L.

Data extracted from the EMR allowed us to analyze the parameters associated to the development of thrombocytopenia. These parameters were partly chosen based on previous publications expounding linezolid thrombocytopenia [14,15,16,17,25,36] and on available data collected in the medical record (see all parameters tested in Table S5 (Supplementary Materials)).

Statistical analyses were performed using IBM^®^ SPSS^®^ statistics version 26.0 (IBM^®^, Armonk, NY, USA). Distribution and normality of quantitative variables were assessed based on Kolmogorov-Smirnov test. As all variables tested were not normally distributed, data were expressed as median and inter-quartile range. Data were compared between groups using the non-parametric Mann-Whitney U test. Categorical variables were compared between groups using Pearson’s Chi-squared test or Fisher exact test according to the condition of validity of each test. The analysis of parameters associated with the development of thrombocytopenia was performed on the subset of patients for whom hemogram data were available. A univariate analysis and a multivariable logistic regression analysis were used to identify determinants associated with the development of thrombocytopenia. All variables with a *p* value < 0.2 in the univariate analysis were included in the multivariable analysis. A backward procedure was then performed to select the final multivariate model. The goodness-of-fit of the final model was evaluated thanks to the Hosmer-Lemeshow test. Results of the model were expressed using adjusted odds ratios (OR) 95% confidence interval. Finally, the median time of onset of thrombocytopenia was assessed through a Kaplan-Meier analysis. Non-parametric Mann-Whitney U test was performed to compare the time of onset in different groups of patients.

## Figures and Tables

**Table 1:** Patients’ baseline characteristics.

Patient Characteristics	N (%) or Median (Range)
Patients	230
Treatment	248
Male	93 (37.1)
Age (years)	65 (21–95)
Weight (kg)	76 (34–178)
Body mass index (kg/m^2^)	25 (15–48)
Glomerular filtration rate ^1^ (mL/min)	57 (10–196)
GFR ≥ 60 mL/min	102
30 mL/min ≤ GFR < 60 mL/min	107
GFR < 30 mL/min	10
Charlson index	3 (0–11)
Inpatients/Outpatients	163 (66)/85 (34)
ICU ^2^ patients ^3^	92 (56.4)

^1^ Estimated with MDRD, ^2^ Intensive Care Unit, ^3^ Proportion among inpatients.

**Table 2:** Infections treated, microorganisms, and treatment duration.

Infection Treated ^1^	Number (%)	Median Treatment Duration (Range)	Microorganisms	Number = 257 (%)
SSTI ^2^	31 (12.5)	19.5 (1–72)	*E. faecium*	66 (25.7)
Pneumonia	18 (7.2)	7 (1–16)	MRSE ^2^	61 (23.7)
Primary bacteremia ^3^	33 (13.3)	8 (2–52)	MRSA ^2^	45 (17.5)
Secondary bacteremia ^3^	43 (17.4)	7 (1–29)	VRE ^2^	24 (9.3)
Bone & joint infections	33 (13.3)	3 (7–90)	MSSA ^2^	12 (4.7)
Gastrointestinal infections	21 (8.5)	8 (2–44)	*E. faecalis*	11 (4.3)
Medical device infections	15 (6)	10 (1–30)	MSSE ^2^	2 (0.8)
Urinary tract infections	13 (5.2)	8 (1–21)	Others ^4^	36 (14)
Mediastinitis	9 (3.6)	11 (5–28)		
Endocarditis	5 (2)	30 (7–45)		
CNS ^2^ infections	5 (2)	10 (4–14)		
MRSA ^2^ decolonization	5 (2)	7.5 (4–20)		
Others	17 (7)			

^1^ Infections classification inspired by the Global PPS project https://www.global-pps.com/project/, (accessed on 3 May 2021). ^2^ Acronyms: SSTI, skin and soft tissue infection; CNS, central nervous system; MRSA, methicillin resistant *staphylococcus aureus*; MRSE, methicillin resistant *staphylococcus epidermidis*; VRE, vancomycin resistant *Enterococcus*; MSSA, methicillin sensitive *Staphylococcus aureus*; MSSE, methicillin sensitive *Staphylococcus epidermidis*. ^3^ Primary bacteremia: No other source identified; Secondary bacteremia: Associated to another source of infection. ^4^ Diverse linezolid-susceptible species, including other Staphylococci, Streptococci, Corynebacteria, non-tuberculosis Mycobacteria).

**Table 3:** Adverse drug reactions reported in medical records and/or identified from laboratory data and their corresponding Naranjo score.

Type of Adverse Drug Reactions	N (%)	Naranjo Score Median (Range)	N of ADR with a Naranjo Score ≥ 5 (%)
Thrombocytopenia	43 (18.9)	4 (1–7)	15 (34.9)
Anemia	17 (6.8)	4 (0–7)	6 (46.2)
Gastrointestinal disorders	13 (5.2)	4 (2–7)	4 (30.8)
Peripheral neuropathy	4 (1.6)	4 (3–5)	2 (50)
Lactic acid serum level > 2.2 mmol/L	4 (1.6)	4 (0–5)	1 (25)
Paresthesia	4 (1.6)	3 (3–6)	1 (25)
Skin disorders	3 (1.2)	1.5 (0–3)	0
Fatigue	3 (1.2)	5 (0–6)	2 (40)
Neutropenia	2 (0.8)	4	2 (100)
Leucopenia ^1^	2 (0.8)	2.5 (1–4)	0
Renal failure ^2^	1 (0.8)	4	0
Taste alteration	1 (0.4)	6	1 (100)
Suspicion of serotonin syndrome	1 (0.4)	4	0
SIADH ^3^	1 (0.4)	3	0

^1^ Leucocytes < 4 × 10^3^/mm^3^ (1.1 × 10^3^/mm^3^–1.9 × 10^3^/mm^3^). ^2^ Creatinine level > 1.4 mg/dL (3.8 mg/dL). ^3^ SIADH, Syndrome of Inappropriate Antidiuretic Hormone.

**Table 4:** Univariate and multivariate analysis of the parameters associated with the development of thrombocytopenia.

Parameters	Univariate Analysis OR [95% CI]	*p*-Value	Multivariate Analysis Adjusted OR [95% CI]	*p*-Value
Inpatients	0.598 [0.3–1.192]	0.144		
Charlson index ≥ 4	2.218 [1.088–4.522]	0.005	2.534 [1.128–5.694]	0.024
Diabetes	2.065 [1.043–4.087]	0.037		
Renal function (<60 mL/min)	2.088 [1.015–4.294]	0.045	3.694 [1.649–8.275]	0.01
Treatment duration > 10 days	5.687 [2.501–12.933]	0.000	7.944 [3.197–19.738]	<0.001

## Data Availability

Data are available from the authors upon request.

## Supplementary Materials

The following are available online at https://www.mdpi.com/article/10.3390/antibiotics10050530/s1. Figure S1: Distribution of the number of infections treated with linezolid among the four included hospital centers. Figure S2: Distribution of the isolated microorganisms per type of infection. Table S1: Characteristics of inpatients and outpatients. Table S2: Characteristics of patients with or without thrombocytopenia. Table S3: Frequency of ADR in this study as compared to the incidence reported in the SmPC or the US label. Table S4: Comorbidities included in Charlson comorbidity index and identified in patients with thrombocytopenia. Table S5: Parameters tested as possibly associated with the development of thrombocytopenia.
