# Supplementary material for: Clinical Use and Adverse Drug Reactions of Linezolid: A Retrospective Study in Four Belgian Hospital Centers
Source: Antibiotics (Basel). 2021 May 4;10(5):530. doi: 10.3390/antibiotics10050530 (PMC8147790; doi:10.3390/antibiotics10050530)
Supplement: Supplementary file 1 [file antibiotics-10-00530-s001.zip › antibiotics-1208367-supplementary.pdf]

antibiotics

Clinical Use and Adverse Drug Reactions of Linezolid: A Retrospective Study in Four Belgian Hospital Centers

Hélène Thirot, Caroline Briquet, Frédéric Frippiat, Frédérique Jacobs, Xavier Holemans, Séverine Henrard, Paul M. Tulkens, Anne Spinewine, Françoise Van Bambeke

Supplementary Material

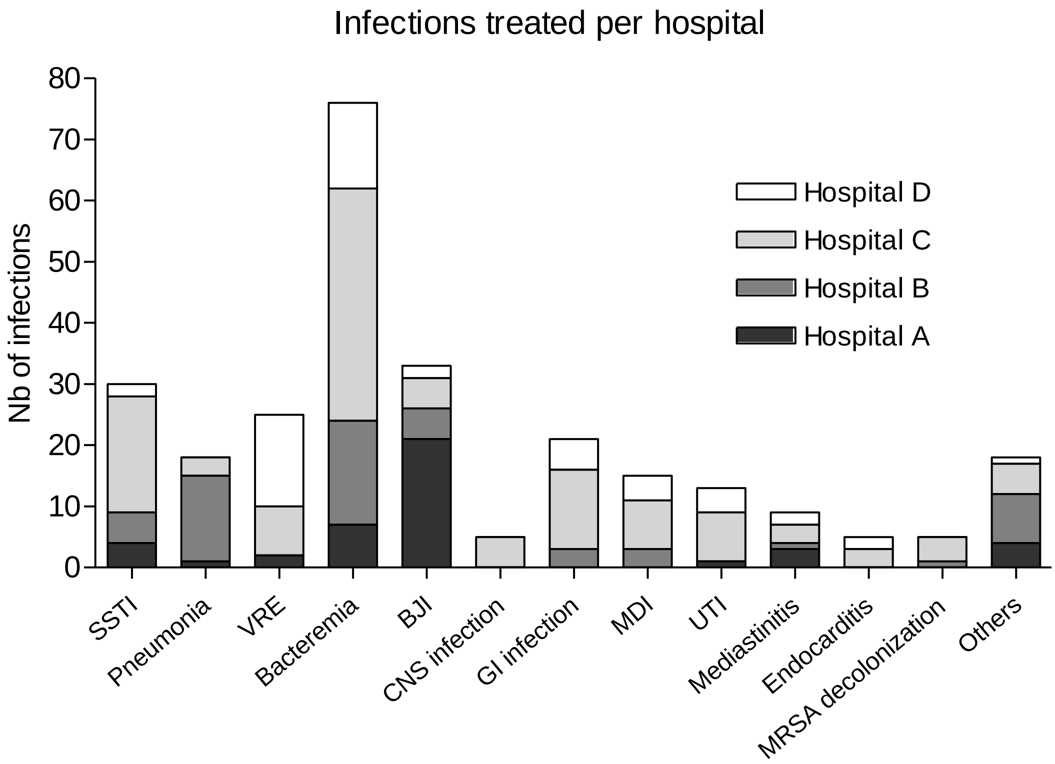

Figure S1: Distribution of the number of infections treated with linezolid among the 4 included hospital centers.

BJI, bone and joint infection; MDI, medical device infection; SSTI, skin and soft tissue infection; UTI, urinary tract infection; VRE, vancomycin-resistant Enterococcus. Each hospital is identified by a letter.

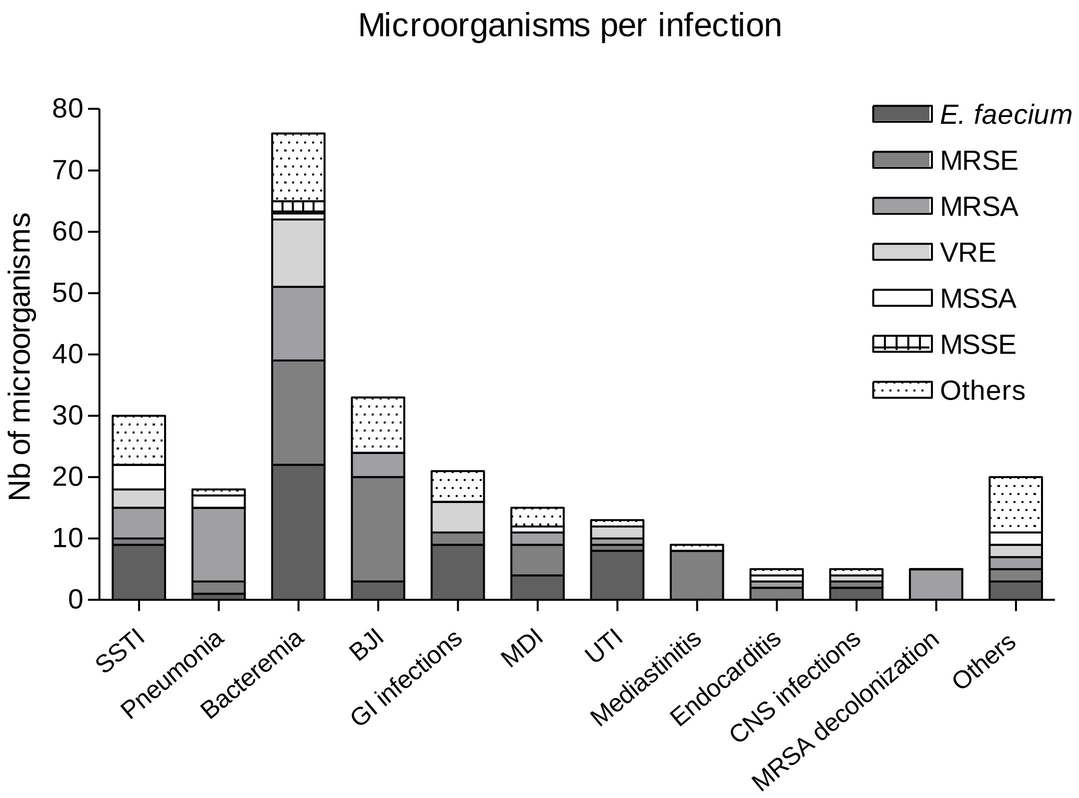

Figure S2: Distribution of the isolated microorganisms per type of infection.
BJI, bone and joint infection; CNS, central nervous system, MDI, medical infection; MRSA, methicillin resistant *Staphylococcus aureus*; MRSE, methicillin resistant *Staphylococcus epidermidis*; MSSA, methicillin sensitive *S. aureus*; MSSE, methicillin sensitive *S. epidermidis;* SSTI, skin and soft tissue infection; UTI, urinary tract infection.

Table S1. Characteristics of inpatients and outpatients

Parameters	Inpatients median (range) or nb (%)	Outpatients median (range) or nb (%)	P-value [1]
Renal function (GFR ml/min)	46.5 (10-196)	89 (15 – 195)	**0.000**
Treatment duration (days)	7 (1 – 52)	21 (2 – 90)	**0.000**
Basal platelet level	203 (1 – 1039)	297 (4 – 624)	**0.000**
Charlson index	2 (0 – 9)	2 (0-8)	0.059
Thrombocytopenia	26 (60.5)	17 (39.5)	0.142
Anemia	10 (58.8)	7 (41.2)	0.309

[1] Values in bold denote statistically significant differences ($p< 0.05$)

Table S2. Characteristics of patients with or without thrombocytopenia

Parameters	No Thrombocytopenia (n = 185) N (%) or median (range)	Thrombocytopenia (n = 43) N (%) or median (range)	P-value [3]
Male (%)	62.7	65.1	0.768
Age (year)	65 (21-95)	67 (26-86)	0.495
Weight (kg)	75 (34-178)	79 (48-132)	0.585
Body mass index (kg/m^2)	25.5 (15-47)	25.9 (16-48)	0.785
INpatients/OUTpatients	133/52	26/17	0.142
Diabetes (%)	51 (27.6)	19 (44.2)	**0.035**
Charlson index	3 (0-11)	4 (0-9)	**0.003**
GFR (ml/min)	58 (11-173)	46 (10-196)	0.087
Renal insufficiency (< 60 ml/min)	84 (52.5)	30 (69.8)	**0.043**
Treatment duration (days)	8 (1-90)	17 (3-53)	**0.000**
Basal platelet count (x10^3/ml)	243 (1-1039)	196 (29 – 507)	0.267
Previous treatment with vancomycin (%)	100 (54.1)	26 (60.5)	0.446
Route of administration (IV/PO)	81/104	19/24	0.962
Dose/Kg/Day	16 (6.7-35.3)	15.2 (9.1-25)	0.584
Isolated microorganism [1]			
MRSA (%)	29 (15.7)	8 (18.6)	0.639
VRE (%)	17 (9.2)	6 (14)	0.300
Staphylococcus aureus (%)	8 (4.3)	4 (9.3)	0.188
Enterococcus faecium (%)	55 (29.7)	10 (15.4)	0.397
Staphylococcus epidermidis (%)	50 (27)	8 (18.6)	0.390
Type of infection [2]			
Pneumonia (%)	13 (7)	1 (2.3)	0.247
SSTI (%)	20 (10.8)	8 (18.6)	0.161
Bone and joint infections (%)	27 (14.5)	4 (9.3)	0.356
Bacteremia (%)	56 (30.2)	13 (30.2)	0.996
Gastrointestinal infections (%)	15 (5.9)	5 (11.6)	0.469
Urinary tract infections (%)	9 (4.9)	2 (4.7)	0.953
IN/OFF label indication	32/153	8/35	0.839
Comedication			
Rifampin	10 (5.4)	3 (7)	0.715
Amiodarone	13 (7.1)	4 (9.3)	0.539
Amlodipine	23 (12.6)	8 (18.6)	0.301
Omeprazole	53 (29)	9 (20.9)	0.288
Pyridoxine	27 (11.8%)	7 (3.1%)	0.780
Anticancer therapy	15 (8.1%)	3 (7%)	0.797

[1] Only most frequent microorganisms are included in the table

[2] Only most frequent infections are included in the table

[3] Values in bold denote statistically significant differences

Table S3: Frequency of ADR in this study as compared to the incidence reported in the SmPC or the US label

Type of side effect	N (%)	ADR in European SmPC	ADR in US label (%)
Thrombocytopenia	43 (18.9)	≥ 1/1.000 to < 1/100	3 [1]
Anemia	17 (6.8)	≥ 1/100 to < 1/10	7.1 [1]
Gastrointestinal disorders	13 (5.2)	≥ 1/100 to < 1/10	8.3
Peripheral neuropathy	4 (1.6)	Indeterminate	Indeterminate [2]
Lactic acid serum level > 2.2mmol/L	4 (1.6)	Indeterminate	Indeterminate [2]
Paresthesia	4 (1.6)	≥ 1/1.000 to < 1/100	/ [3]
Skin disorders	3 (1.2)	≥ 1/100 to < 1/10	2.3
Fatigue	3 (1.2)	≥ 1/1.000 to < 1/100	1.8
Neutropenia	2 (0.8)	≥ 1/1.000 to < 1/100	1.1 [1]
Leucopenia	2 (0.8)	≥ 1/1.000 to < 1/100	2.2 [1]
Renal failure	1 (0.8)	≥ 1/1.000 to < 1/100	/ [3]
Taste alteration	1 (0.4)	≥ 1/100 to < 1/10	1
Serotonin syndrome	1 (0.4)	Indeterminate	Indeterminate [2]
SIADH	1 (0.4)	Indeterminate	/ [3]

[1] As mentioned in FDA label; defined as less than 75% of lower limit of normal and or/baseline. No significant difference between the comparators and linezolid in phase III comparator-controlled clinical trials.

[2] Frequency of these ADR is not reported in US label as observed in postmarketing experience.

[3] Not mentioned in the SmPC

Table S4: Comorbidities included in Charlson comorbidity index and identified in patients with thrombocytopenia

		Patients with thrombocytopenia (n=43)		
**Comorbidities**	**Total number of patients**	**Number of patients**	**% of the patients with thrombocytopenia**	**% of the patients with the comorbidity**
Myocardial infarction	29	7	16.2	24.1
Congestive heart failure	9	3	7.0	33.3
Peripheral vascular disease	20	6	14.0	30
Cerebrovascular disease	19	4	9.3	21
Dementia	16	4	9.3	25
Chronic pulmonary disease	55	7	16.2	12.7
Ulcer disease	13	4	9.3	30.8
Rheumatologic disease	0	0	0	0
Mild liver disease	16	5	11.6	31.2
Diabetes	78	19	44.2	24.3
Hemiplegia	3	1	2.3	33.3
Moderate or severe renal disease	20	9	20.9	45
Diabetes with chronic complications	30	6	14.0	20
Any tumor	34	8	18.6	23.5
Leukemia	9	0	0	0
Lymphoma	5	1	2.3	20
Moderate to severe liver disease	26	7	16.2	26.9
Metastatic solid tumor	10	2	4.7	20
AIDS	2	0	0	0

Table S5: Parameters tested for the development of thrombocytopenia

Patient		Comedications	Treatment	Infections
Inpatient/Outpatient	Diabetes	Rifampin	Switch from vancomycin	Type of infection
Gender	Cancer	Amiodarone	First-line treatment	In-label/off-label infection
Age	Tobacco	Amlodipine	Route of administration	Isolated microorganism
Weight	Alcohol	Omeprazole	Treatment duration	
Body mass index	GFR	Pyridoxine		
Dose/Kg/day	Creatinine level	Number of comedication		
Charlson index	Basal platelet count			
